# Supplementary material for: Interrogating COVID-19 vaccine intent in the Philippines with a nationwide open-access online survey
Source: PeerJ. 2022 Feb 16;10:e12887. doi: 10.7717/peerj.12887 (PMC8857903; doi:10.7717/peerj.12887)
Supplement: Supplemental Information 2 — Lists of questions in both English and in Filipino that were presented to respondents using a digital online platform. [file peerj-10-12887-s002.pdf]

ENGLISH

DEMOGRAPHIC INFORMATION

1. What is your age?
2. What is your sex? Male or Female
3. What is your marital status? Single or Married
4. What is your highest level of education? Elementary School or Below
   Junior High School
   Senior High School
   College/University or Above
5. What is your occupation? Professional/White Collar Worker
   Blue Collar Worker
   Self-employed
   Student
   Housewife/Retired/
   Unemployed/
   Other
6. Do you live in a rural or urban area? Urban or Rural
7. Which region of the Philippines do you live in?
   I, II, III, IV-A, IV-B, V, CAR, NCR,
   VI, VII, VIII, IX, X, XI, XII, XIII, or BARMM
8. Have you ever tested positive for COVID-19? Yes or No
9. Do you know anyone who has tested positive for COVID-19?
   Yes or No
10. Do you have an existing chronic condition like cancer, cardiovascular disease, diabetes, etc.?
    Yes or No
11. How would you rate your overall health?
    Very Good, Good, Fair, Poor, Very Poor

OPENNESS TO THE COVID-19 VACCINE

12. If a vaccine for COVID-19 is available in the Philippines, would you use it?
    Definitely Yes, Probably Yes, Unsure, Probably No, Definitely No

PREFERENCE FOR THE COVID-19 VACCINES

13. Rate your confidence in using a vaccine for COVID-19 made in China.
    Completely Confident, Confident, Not Confident, Completely Not Confident.

14. Rate your confidence in using a vaccine for COVID-19 made in Russia.
    Completely Confident, Confident, Not Confident, Completely Not Confident.

15. Rate your confidence in using a vaccine for COVID-19 made in the USA or Europe.
    Completely Confident, Confident, Not Confident, Completely Not Confident.

16. Please indicate your preference?
a. I prefer a vaccine made in China.
b. I prefer a vaccine made in Russia.
c. I prefer a vaccine made in the USA or Europe.
d. I do not have a preference. I will use any safe and effective vaccine.

17. If a vaccine was made using fetal cell lines obtained from an aborted baby, would you use it?
    Definitely Yes, Probably Yes, Unsure, Probably No, Definitely No

HEALTH BELIEF MODEL

18. My chance of getting COVID-19 in the next few months is high.
    Strongly Agree, Agree, Disagree, Strongly Disagree

19. I am worried about the likelihood of getting COVID-19.
    Strongly Agree, Agree, Disagree, Strongly Disagree

20. I am worried about the likelihood of someone in my family getting COVID-19.
    Strongly Agree, Agree, Disagree, Strongly Disagree

21. COVID-19 is a serious disease with life-threatening complications.
    Strongly Agree, Agree, Disagree, Strongly Disagree

22. I will be very sick if I get COVID-19.
    Strongly Agree, Agree, Disagree, Strongly Disagree

23. I am afraid of getting COVID-19.
Strongly Agree, Agree, Disagree, Strongly Disagree

24. I am afraid that someone in my family will get COVID-19.
Strongly Agree, Agree, Disagree, Strongly Disagree

VACCINATION BELIEF MODEL

25. Vaccination will decrease my chances of getting COVID-19.
Strongly Agree, Agree, Disagree, Strongly Disagree

26. Vaccination will decrease my worries about catching COVID-19.
Strongly Agree, Agree, Disagree, Strongly Disagree

27. I worry about the possible side-effects of the COVID-19 vaccine.
Strongly Agree, Agree, Disagree, Strongly Disagree

28. I worry about the effectiveness of the COVID-19 vaccine.
Strongly Agree, Agree, Disagree, Strongly Disagree

29. I worry about the safety of the COVID-19 vaccine.
Strongly Agree, Agree, Disagree, Strongly Disagree

30. I worry about the high cost of the COVID-19 vaccine.
Strongly Agree, Agree, Disagree, Strongly Disagree

31. I worry about fake COVID-19 vaccines.
Strongly Agree, Agree, Disagree, Strongly Disagree

32. I worry that the COVID-19 vaccines were made too quickly.
Strongly Agree, Agree, Disagree, Strongly Disagree

33. I worry that the COVID-19 vaccines were not tested properly.
Strongly Agree, Agree, Disagree, Strongly Disagree

34. I worry that the COVID-19 vaccines will make me sick.
Strongly Agree, Agree, Disagree, Strongly Disagree

35. I worry that the COVID-19 vaccines will not be effective against new variants of the virus.
Strongly Agree, Agree, Disagree, Strongly Disagree

36. I do not think that I need the vaccine because I am waiting for herd immunity in the country.
Strongly Agree, Agree, Disagree, Strongly Disagree

37. I will only receive the COVID-19 vaccine after many other people receive the vaccine.
Strongly Agree, Agree, Disagree, Strongly Disagree

38. I will only receive the COVID-19 vaccine after politicians receive the vaccine.
Strongly Agree, Agree, Disagree, Strongly Disagree

WILLINGNESS TO PAY

39. What is the maximum amount you are willing to pay for two doses of the COVID-19 vaccine?

PHP 500
PHP 1,000
PHP 1,500
PHP 2,000
PHP 2,500
PHP 3,000
PHP 3,500
PHP 4,000

FILIPINO

DEMOGRAPIKONG IMPORMASYON

1. Ilang taon ka na?
2. Ano ang iyong kasarian? — Lalaki o Babae
3. Ano ang iyong kalagayang sibil? — Single o Kasal
4. Anong pinakamataas na antas ng edukasyon ang iyong natapos?
   - Elementarya o mas mababa
   - Junior High School
   - Senior High School
   - Kolehiyo o mas mataas
5. Ano ang iyong trabaho?
   - Propesyonal (White Collar)
   - Ordinaryong manggagawa (Blue Collar)
   - Self-employed
   - Estudyante
   - Maybahay/Retirado/Walang trabaho/
   - Iba pa.
6. Ikaw ba ay naninirahan sa probinsya o lungsod? — Probinsya o Lungsod
7. Saang rehiyon sa Pilipinas ka nakatira?
   I, II, III, IV-A, IV-B, V, CAR, NCR,
   VI, VII, VIII, IX, X, XI, XII, XIII, BARMM
8. Nagpositibo ka na ba sa COVID-19? — Oo o Hindi
9. May kakilala ka bang nagpositibo sa COVID-19? — Meron o Wala
10. Kasalukuyan ka bang mayroong malalang kalagayan tulad ng kanser, sakit sa puso, diabetes, o iba pa? — Meron o Wala

11. Paano mo mailalarawan ang kalagayan ng iyong kalusugan?
    Napakabuti, Mabuti, Katamtaman, Masama, Napakasama

PAGKABUKAS NG ISIPAN SA BAKUNA LABAN SA COVID-19

12. Kung mayroon na sa Pilipinas ng bakuna laban sa COVID-19, ikaw ba ay magpapabakuna?
    Siguradong oo, Marahil na oo, Hindi ako sigurado, Marahil na hindi, Siguradong hindi

PERSONAL NA KAGUSTUHAN SA BAKUNA LABAN SA COVID-19

13. Ilarawan ang iyong pagtitiwala sa paggamit ng bakuna laban sa COVID-19 na gawa sa Tsina.
    Labis na nagtitiwala, Nagtitiwala, Hindi nagtitiwala, Labis na hindi nagtitiwala

14. Ilarawan ang iyong pagtitiwala sa paggamit ng bakuna laban sa COVID-19 na gawa sa Russia.
Labis na nagtitiwala, Nagtitiwala, Hindi nagtitiwala, Labis na hindi nagtitiwala

15. Ilarawan ang iyong pagtitiwala sa paggamit ng bakuna laban COVID-19 na gawa sa Amerika o Europa.
Labis na nagtitiwala, Nagtitiwala, Hindi nagtitiwala, Labis na hindi nagtitiwala

16. Paki-saad kung alin sa mga sumusunod ang iyong mas gusto.
   a. Mas gusto ko ang bakunang gawa sa Tsina.
   b. Mas gusto ko ang bakunang gawa sa Russia.
   c. Mas gusto ko ang bakunang gawa sa Amerika o Europa.
   d. Kahit ano, basta ang bakuna ay ligtas at epektibo

17. Kung mayroong bakuna na gawa sa pinalaglag na sanggol, gagamitin mo ba ito?
Siguradong oo, Marahil na oo, Hindi ako sigurado, Marahil na hindi, Siguradong hindi

MODELO NG PANINIWALA SA KALUSUGAN

18. Mataas ang posibilidad na mahawaan ako ng COVID-19 sa mga susunod na buwan
Lubos na sumasang-ayon, Sumasang-ayon, Hindi sumasang-ayon, Lubos na hindi sumasang-ayon

19. Ako ay nag-aalala sa posibilidad na magkaroon ako ng COVID-19.
Lubos na sumasang-ayon, Sumasang-ayon, Hindi sumasang-ayon, Lubos na hindi sumasang-ayon

20. Ako ay nag-aalala sa posibilidad na may magkaroon ng COVID-19 sa aking pamilya.
Lubos na sumasang-ayon, Sumasang-ayon, Hindi sumasang-ayon, Lubos na hindi sumasang-ayon

21. Ang COVID-19 ay isang seryosong karamdaman na may hatid na mga komplikasyong may banta sa buhay.
Lubos na sumasang-ayon, Sumasang-ayon, Hindi sumasang-ayon, Lubos na hindi sumasang-ayon

22. Ako ay lubhang magkakasakit kung mahawa ako ng COVID-19.
Lubos na sumasang-ayon, Sumasang-ayon, Hindi sumasang-ayon, Lubos na hindi sumasang-ayon

23. Ako ay natatakot magkaroon ng COVID-19.
Lubos na sumasang-ayon, Sumasang-ayon, Hindi sumasang-ayon, Lubos na hindi sumasang-ayon

24. Ako ay natatakot na mayroong magka-COVID-19 sa aking pamilya.
Lubos na sumasang-ayon, Sumasang-ayon, Hindi sumasang-ayon, Lubos na hindi sumasang-ayon

MODELO NG PANINIWALA SA BAKUNA

25. Mababawasan ang posibilidad na mahawaan ako ng COVID-19 kapag nasimulan na ang pagbabakuna laban dito.
Lubos na sumasang-ayon, Sumasang-ayon, Hindi sumasang-ayon, Lubos na hindi sumasang-ayon

26. Mababawasan ang aking pagkabahala na mahawaan ako ng COVID-19 kapag nagsimula na ang pagbabakuna.
Lubos na sumasang-ayon, Sumasang-ayon, Hindi sumasang-ayon, Lubos na hindi sumasang-ayon

27. Ako ay nag-aalala sa posibleng *side effects* ng bakuna laban sa COVID-19.
Lubos na sumasang-ayon, Sumasang-ayon, Hindi sumasang-ayon, Lubos na hindi sumasang-ayon

28. Ako ay nag-aalala sa bisa ng bakuna laban sa COVID-19.
Lubos na sumasang-ayon, Sumasang-ayon, Hindi sumasang-ayon, Lubos na hindi sumasang-ayon

29. Ako ay nag-aalala sa kaligtasan ng bakuna laban sa COVID-19.
Lubos na sumasang-ayon, Sumasang-ayon, Hindi sumasang-ayon, Lubos na hindi sumasang-ayon

30. Ako ay nag-aalala sa mataas na halaga ng bakuna laban sa COVID-19.
Lubos na sumasang-ayon, Sumasang-ayon, Hindi sumasang-ayon, Lubos na hindi sumasang-ayon

31. Ako ay nag-aalala sa mga pekeng bakuna laban sa COVID-19.
Lubos na sumasang-ayon, Sumasang-ayon, Hindi sumasang-ayon, Lubos na hindi sumasang-ayon

32. Ako ay nag-aalala na masyadong naging mabilis ang paggawa sa mga bakuna laban sa COVID-19.
Lubos na sumasang-ayon, Sumasang-ayon, Hindi sumasang-ayon, Lubos na hindi sumasang-ayon

33. Ako ay nag-aalala na ang mga bakuna laban sa COVID-19 ay hindi dumaan sa masusing pagsusuri.
Lubos na sumasang-ayon, Sumasang-ayon, Hindi sumasang-ayon, Lubos na hindi sumasang-ayon

34. Ako ay nag-aalala na maaari akong magkasakit pagkatapos kong mabakunahan laban sa COVID-19.
Lubos na sumasang-ayon, Sumasang-ayon, Hindi sumasang-ayon, Lubos na hindi sumasang-ayon

35. Ako ay nag-aalala na ang kasalukuyang mga bakuna laban sa COVID-19 ay hindi magiging epektibo laban sa mga bagong *strain* ng virus na ito.
Lubos na sumasang-ayon, Sumasang-ayon, Hindi sumasang-ayon, Lubos na hindi sumasang-ayon

36. Sa tingin ko ay hindi ko kailangan magpabakuna sapagkat hinihintay ko lamang na makamit ng bansa ang *herd immunity* kung saan ang karamihan sa populasyon ay ligtas at hindi na tatamaan ng karamdamang ito.
Lubos na sumasang-ayon, Sumasang-ayon, Hindi sumasang-ayon, Lubos na hindi sumasang-ayo

37. Magpapabakuna lamang ako kapag maraming tao na ang tapos magpa-bakuna laban sa COVID-19.
Lubos na sumasang-ayon, Sumasang-ayon, Hindi sumasang-ayon, Lubos na hindi sumasang-ayon

38. Magpapabakuna lamang ako pagkatapos magpabakuna ng mga pulitiko laban sa COVID-19.
Lubos na sumasang-ayon, Sumasang-ayon, Hindi sumasang-ayon, Lubos na hindi sumasang-ayon

KAHANDAANG MAGBAYAD

39. Ano ang pinakamalaking halagang handa mong ibayad para sa dalawang dosis ng bakuna laban sa COVID-19?

PHP 500
PHP 1,000
PHP 1,500
PHP 2,000
PHP 2,500
PHP 3,000
PHP 3,500
PHP 4,000
