# Supplementary material for: Interrogating COVID-19 vaccine intent in the Philippines with a nationwide open-access online survey
Source: PeerJ. 2022 Feb 16;10:e12887. doi: 10.7717/peerj.12887 (PMC8857903; doi:10.7717/peerj.12887)
Supplement: Supplemental Information 3 [file peerj-10-12887-s003.pdf]

Interrogating COVID-19 Vaccine Hesitancy in the Philippines with a Nationwide Open-Access Online Survey Nominal Data Codebook

- Sex_recoded
  - 1 = Male
  - 2 = Female

- Martial_Status_recoded_Q51
  - 1 = Single
  - 2 = Married

- Education_recoded_Q52
  - 1 = Elementary School or Below
  - 2 = Junior High School
  - 3 = Senior High School
  - 4 = College/University or Above

- Occupation_recoded_Q53
  - 1 = Blue Collar Worker
  - 2 = Professional/White Collar Worker
  - 3 = Self-employed
  - 4 = Student
  - 5 = Housewife/Retired/Unemployed/Other

- Income_recoded_Q106
  - 1 = PHP 10,000 or less
  - 2 = PHP 10,000 to 20,000
  - 3 = PHP 20,000 to 100,000
  - 4 = PHP 100,000 or more

- AreaRural_Urban_recoded_Q54
  - 1 = Rural
  - 2 = Urban

- Live_in_QuezonCity_recoded_Q56
  - 1 = Yes
  - 2 = No

- Test_Pos_COVID_Q57
  - 1 = Yes
  - 2 = No

- Know_Someone_Test_Pos_COVID_Q58
    - 1 = Yes
    - 2 = No

- Chronic_Condition_Q59
    - 1 = Yes
    - 2 = No

- Overall_Health_Q60
    - 1 = Very Poor
    - 2 = Poor
    - 3 = Fair
    - 4 = Good
    - 5 = Very Good

- Preference_Where_Vax_Made_Q66
    - 1 = do not have a preference. I will use any safe and effective vaccine.
    - 2 = I prefer a vaccine made in China.
    - 3 = I prefer a vaccine made in Russia.
    - 4 = I prefer a vaccine made in the USA or Europe.

- Would_Use_Fetal_Cell_COVID_Vax_Q65
    - 1 = Definitely NO
    - 2 = Probably No
    - 3 = Unsure
    - 4 = Probably Yes
    - 5 = Definitely Yes
    - 6 = Siguradong Yes

- WTP_Recode
    - 1 = PHP 500–1000
    - 2 = PHP 1500–2500
    - 3 = PHP 3000–4000
